# Predictors of the final place of care of patients with advanced cancer receiving integrated home-based palliative care: a retrospective cohort study

**DOI:** 10.1186/s12904-021-00865-5

**Published:** 2021-10-18

**Authors:** Ri Yin Tay, Rozenne W. K. Choo, Wah Ying Ong, Allyn Y. M. Hum

**Affiliations:** 1 Dover Park Hospice, 10 Jalan Tan Tock Seng, Singapore, 308436 Singapore; 2 The Palliative Care Centre for Excellence in Research and Education, Singapore, Singapore; 3 Department of Palliative Medicine, Tan Tock Seng Hospital, Singapore, Singapore

**Keywords:** Final place of care, Place of death, Advanced cancer, Integrated, Home-based palliative care

## Abstract

**Background:**

Meeting patients’ preferences for place of care at the end-of-life is an indicator of quality palliative care. Understanding the key elements required for terminal care within an integrated model may inform policy and practice, and consequently increase the likelihood of meeting patients’ preferences. Hence, this study aimed to identify factors associated with the final place of care in patients with advanced cancer receiving integrated, home-based palliative care.

**Methods:**

This retrospective cohort study included deceased adult patients with advanced cancer who were enrolled in the home-based palliative care service between January 2016 and December 2018. Patients with < 2 weeks’ enrollment in the home-based service, or ≤ 1-week duration at the final place of care, were excluded. The following information were retrieved from patients’ electronic medical records: patients’ and their families’ characteristics, care preferences, healthcare utilization, functional status (measured by the Palliative Performance Scale (PPSv2)), and symptom severity (measured by the Edmonton Symptom Assessment System). Multivariate logistic regression was employed to identify independent predictors of the final place of care. Kappa value was calculated to estimate the concordance between actual and preferred place of death.

**Results:**

A total of 359 patients were included in the study. Home was the most common (58.2%) final place of care, followed by inpatient hospice (23.7%), and hospital (16.7%). Patients who were single or divorced (OR: 5.5; 95% CI: 1.1–27.8), or had older family caregivers (OR: 3.1; 95% CI: 1.1–8.8), PPSv2 score ≥ 40% (OR: 9.1; 95% CI: 3.3–24.8), pain score ≥ 2 (OR: 3.6; 95% CI: 1.3–9.8), and non-home death preference (OR: 23.8; 95% CI: 5.4–105.1), were more likely to receive terminal care in the inpatient hospice. Patients who were male (OR: 3.2; 95% CI: 1.0–9.9), or had PPSv2 score ≥ 40% (OR: 8.6; 95% CI: 2.9–26.0), pain score ≥ 2 (OR: 3.5; 95% CI: 1.2–10.3), and non-home death preference (OR: 9.8; 95% CI: 2.1–46.3), were more likely to be hospitalized. Goal-concordance was fair (72.6%, kappa = 0.39).

**Conclusions:**

Higher functional status, greater pain intensity, and non-home death preference predicted institutionalization as the final place of care. Additionally, single or divorced patients with older family caregivers were more likely to receive terminal care in the inpatient hospice, while males were more likely to be hospitalized. Despite being part of an integrated care model, goal-concordance was sub-optimal. More comprehensive community networks and resources, enhanced pain control, and personalized care planning discussions, are recommended to better meet patients’ preferences for their final place of care. Future research could similarly examine factors associated with the final place of care in patients with advanced non-cancer conditions.

**Supplementary Information:**

The online version contains supplementary material available at 10.1186/s12904-021-00865-5.

## Background

Providing quality palliative care is of paramount importance in relieving the suffering, and improving the quality of life of patients facing life-threatening illnesses [1]. Meeting patients’ preferences for place of death is an indicator of quality palliative care [2]. This requires an awareness of patients’ and their families’ preferences, often established through care planning discussions [3]. Although many individuals prefer to be cared for, and die in the comfort and familiarity of their homes [4, 5], most in Asia and Europe do not do so [6, 7]. A systematic review classified predictors of place of death in patients with advanced cancer into three broad categories: individual, illness, and environment-related factors. Preference for home death, low functional status, staying with relatives, extended family support, as well as availability and intensity of homecare services, such as home-based palliative care, were strongly associated with home death [8]. Besides providing support for patients’ physical and psychosocial needs, home-based palliative care coordinates referrals to support services. This reduces unnecessary care setting transitions at the end-of-life [9], thereby increasing the likelihood of home death [10, 11].

However, despite it being the preference of many, home death may not be suitable for everyone [12–15]. Inadequately controlled symptoms and acute reversible events may require management in hospitals [4, 11, 14, 16–20]. Some patients may also be reluctant to burden families with their care at home [4, 5, 16, 21], due to the substantial opportunity and societal costs involved [20, 22, 23]. The quality and sustainability of care at home may be affected [8] when the dying trajectory is protracted, and care demands increase beyond families’ capacity to cope [13, 24]. Additionally, there may be cultural and religious misgivings about dying at home, as well as concerns about the possible traumatic effects on children [4].

An integrated palliative care model that coordinates the key services necessary for comprehensive patient care can prevent care fragmentation when unavoidable care setting transitions occur [25, 26]. Such a model, based on a collaboration among a home-based specialist palliative care team, a tertiary hospital, and an inpatient hospice, was established in Singapore in 2012 [26]. This model of care facilitates seamless patient transitions by activating direct admissions to the hospital and inpatient hospice quickly when required. Additionally, the variety of care settings within the model provides patients with the option to choose where they want to be cared for, and die [24, 26]. Patients’ medical records are accessible by all healthcare professionals involved in their care, thereby ensuring care continuity even as they transit between settings. Furthermore, the home-based team regularly reviews patients, and establishes their care preferences early through care planning discussions with them and their families. This minimizes divergence from patients’ expressed wishes during health crises [26].

However, evidence examining factors associated with the place of death of patients within an integrated care model is scarce [27, 28]. Most studies tended to evaluate the model’s effectiveness in facilitating home death instead [20, 29–31]. Existing literature identifying place of death predictors also usually differentiated outcomes based on the setting’s care focus [10] or institutional nature [13, 24, 32]. Additionally, exploring factors associated with the final place of care (defined as the place where patients were cared for, for more than a week, before dying) may be more meaningful [33], as healthcare setting transitions which occurred in the last week of life could negatively affect the care quality of patients with advanced cancer [34]. However, the focus of existing literature has been on the place of death, rather than the final place of care [8, 10, 11, 13, 24, 32]. Gaining an understanding of the elements and infrastructure required to care for patients at the end-of-life in the different settings within the integrated care model may inform practice and policy, and improve the likelihood of meeting patients’ preferences. Hence, the primary aim of this study was to identify factors associated with the final place of care in patients with advanced cancer receiving home-based palliative care within an integrated care model. Additionally, goal-concordance, whether the care delivered was congruent with patients’ preferences, is an important care quality outcome that is rarely evaluated [3]. Studies have shown that families’ support for patients’ preferences is vital to achieving goal-concordant care [8]. Hence, the secondary aims were to examine (i) goal-concordance, and (ii) the congruence between patients’ and their families’ preferences for places of care and death.

## Methods

This was a retrospective, cohort study. Ethics approval was obtained from the local institutional review board. Informed consent was waived as the study involved deceased patients.

### Patient population

Deceased, adult (aged ≥21 years) patients with advanced cancer who were enrolled in the home-based palliative care service from January 2016 to December 2018 were included. Patients whose enrollment duration in the home-based palliative care service was less than two weeks, or who spent less than a week at the final place of care, were excluded.

### Independent variables

Independent variables, including patient sociodemographic, clinical factors, family caregiver characteristics, preferences for places of care and death, healthcare utilization, functional status and symptom severity assessed within two weeks prior to death, or admission to the final place of care, were selected based on a review of existing literature on the topic [8, 10, 11, 13, 24]. These information were extracted from the home-based palliative care service electronic medical records. Charlson Comorbidity Index, a validated prognostic scale consisting of 19 categories weighted from one to six points, was used to capture the number and severity of patients’ comorbidities. Higher scores indicate poorer survival [35]. Functional status had been assessed using the validated Palliative Performance Scale v2 (PPSv2; score range: 0% (death) - 100% (normal function)) by the home-based team during routine home visits [36, 37]. Similarly, symptom severity had been assessed using a psychometrically tested tool commonly used in palliative care, the Edmonton Symptom Assessment System Revised (ESASr; score range: 0 (no symptom) to 10 (worst)), during the home visits. Overall symptom burden was obtained by summing individual symptom scores [38, 39]. Preferences for places of care and death were extracted from the discussions documented in patients’ advance care plan forms and medical records.

### Dependent variable

The dependent variable was the final place of care: home, inpatient hospice, or hospital, which are the three care settings within the integrated care model [26].

### Other data

Information pertaining to reasons for admission to the inpatient hospice or hospital as the final place of care were also extracted to augment the quantitative outcomes. However, the qualitative analysis for this will be undertaken and reported separately. Additionally, information on post-bereavement measures such as families’ acceptance of death, and the bereavement support required, were extracted.

### Data source

All the data were routine clinical documentation obtained from the electronic medical records of the home-based palliative care service.

### Statistical analysis

Variables were summarized using descriptive statistics. Continuous variables were reported as median with interquartile range and mean with standard deviation while categorical variables were reported as frequencies with percentages.

#### Factors associated with the final place of care

Associations between the final place of care (home, inpatient hospice, or hospital) and continuous independent variables were investigated using the Kruskal-Wallis test, while associations with categorical variables were examined using chi-square test. Post-hoc analysis was performed to determine pairs of groups that were significantly different from each other. With three pair-wise comparisons, the *p*-value was adjusted to 0.017 with Bonferroni correction to control for Type 1 error.

Statistically significant variables from post-hoc analysis were then shortlisted for multivariate regression. Variables with high collinearity, categorical variables with low frequencies, symptom scores that were too low for meaningful interpretation, and variables with overlapping concepts, were excluded.

For ease of interpretation and applicability, continuous variables were transformed into categorical variables based on their median values, while multi-categorical variables were collapsed into two categories based on conceptual similarities (Additional file 1: Appendix Tables A1 and A2).

Multivariate binomial models were initially run separately to predict inpatient hospice and hospital as the final place of care with home as the reference category. Statistically significant variables were then entered into multinomial models to identify factors independently associated with the final place of care. The Hosmer-Lemeshow test was used to assess the goodness-of-fit of the models. Predictive accuracy, clinical relevance and Akaike and Bayesian Information Criteria further guided model selection [40].

#### Goal-concordance and congruence

Goal-concordance and congruence between patients’ and their families’ preferences were determined using the Kappa measure of agreement. A Kappa value < 0.2 indicates slight agreement, while values ranging from 0.2–0.4, 0.4–0.6 and 0.6–0.8 indicate fair, moderate, and substantial agreement, respectively. A value > 0.8. denotes almost perfect agreement [41].

All statistical analyses were performed using SPSS, version 25 (IBM Corp, New York). All tests were 2-sided, with the level of statistical significance set at *p* < 0.05 except in cases with Bonferroni adjustment.

## Results

### Background characteristics

**Fig. 1:**
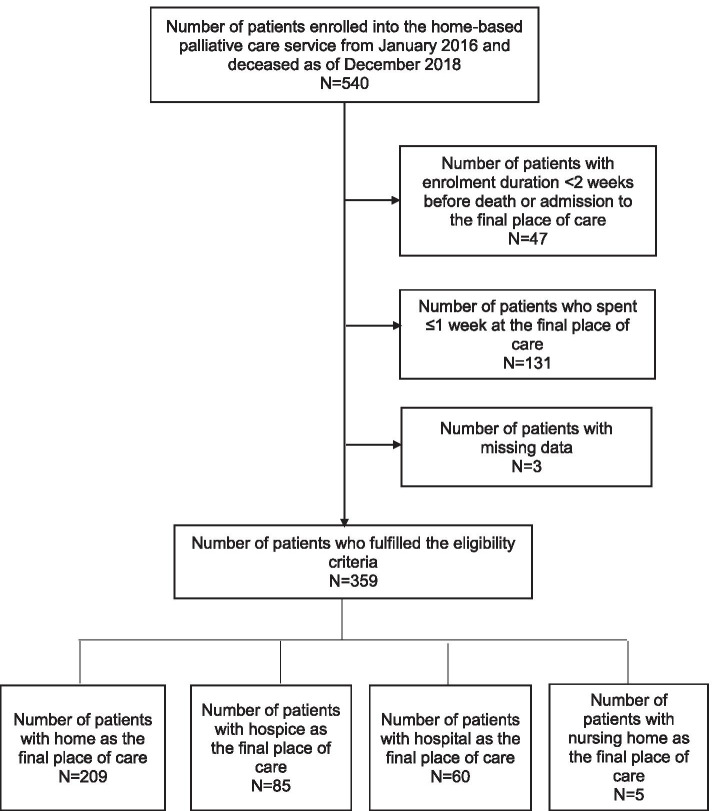
Flowchart of patients

A total of 540 patients with advanced cancer who were enrolled in the home-based palliative care service passed away between January 2016 and December 2018. Of this, 181 patients were excluded for the following reasons: enrollment duration < 2 weeks (*N* = 47); ≤1 week spent at the final place of care (*N* = 131); and, missing data (*N* = 3). The background characteristics of the 359 included patients are shown in Table 1. Home was the most common final place of care (58.2%), followed by inpatient hospice (23.7%), hospital (16.7%), and nursing home (1.4%) (Fig. 1). Duration of stay in the final place of care was significantly longer among patients whose final place of care was home [median (IQR) = 40 (25–84) days], compared to those whose final place of care was the inpatient hospice [median (IQR) = 20 (11–46) days] or hospital [median (IQR) = 16.5 (12–25.5) days] (*p* < 0.0001).

**Table 1:** Comparison of background characteristics of included and excluded patients

Variables	Included patients (***N*** = 359)	Excluded patients (***N*** = 181)	P-value
Age (years)			
Median (IQR)	77 (67–84)	71 (63–80)	< 0.0001
Mean (SD)	75.3 (11.7)	71.2 (11.8)	
Gender			
Male	184 (51.3)	113 (62.4)	0.018
Female	175 (48.7)	68 (37.6)	
Ethnicity			
Chinese	312 (86.9)	169 (93.4)	0.027
Malay	23 (6.4)	2 (1.1)	
Indian	17 (4.7)	6 (3.3)	
Eurasian	3 (0.8)	0	
Others	4 (1.1)	4 (2.2)	
Marital status			
Single	32 (8.9)	24 (13.3)	0.028
Married	178 (49.6)	102 (56.4)	
Divorced/separated	20 (5.6)	12 (6.6)	
Widowed	129 (35.9)	43 (23.8)	
Cancer type			
Locally advanced/non-metastatic	77 (21.4)	22 (12.2)	0.012
Metastatic	282 (78.6)	159 (87.8)	
Cancer site			
Brain	9 (2.5)	1 (0.6)	0.110
Head and neck	13 (3.6)	6 (3.3)	
Gastrointestinal system	97 (27.0)	38 (21.0)	
Hepatobiliary pancreatic system	65 (18.1)	35 (19.3)	
Breast	25 (7.0)	11 (6.1)	
Gynecological	3 (0.8)	2 (1.1)	
Genitourinary	20 (5.6)	11 (6.1)	
Hematological	14 (3.9)	5 (2.8)	
Prostate	10 (2.8)	8 (4.4)	
Skin	3 (0.8)	2 (1.1)	
Lung	76 (21.2)	48 (26.5)	
Multiple sites	10 (2.8)	3 (1.7)	
Unknown	11 (3.1)	5 (2.8)	
Others	3 (0.8)	6 (3.3)	
Duration of diagnosis (months)			
Median (IQR)	7 (2–18.5)	9 (2–27)	0.357
Mean (SD)	17.4 (28.2)	19.7 (32.6)	
Comorbidities			
Cardiovascular disease	99 (27.6)	49 (27.1)	0.982
Congestive heart failure	13 (3.6)	5 (2.8)	0.787
Connective tissue disease	7 (1.9)	6 (3.3)	0.497
Chronic obstructive pulmonary disease	27 (7.5)	14 (7.7)	1.000
Dementia	44 (12.3)	19 (10.5)	0.646
Diabetes mellitus	129 (36.0)	57 (31.4)	
Uncomplicated	86 (24.0)	39 (21.5)	
With end organ damage	43 (12.0)	18 (9.9)	0.576
Liver disease	27 (7.5)	16 (8.9)	
Mild	8 (2.2)	3 (1.7)	
Moderate-severe	19 (5.3)	13 (7.2)	0.625
Myocardial infarct	47 (13.1)	18 (9.9)	0.357
Peripheral vascular disease	10 (2.8)	1 (0.6)	0.158
Peptic ulcer disease	31 (8.6)	12 (6.6)	0.519
Hemiplegia	33 (9.2)	9 (5.0)	0.119
Moderate-severe chronic kidney disease	59 (16.4)	17 (9.4)	0.037
Leukemia	7 (1.9)	3 (1.7)	1.000
Lymphoma	6 (1.7)	2 (1.1)	0.891
Acquired immune deficiency syndrome	2 (0.6)	0	0.798
Charlson comorbidity index score			
Median (IQR)	11 (9–12)	10 (9–12)	0.153
Mean (SD)	10.7 (2.4)	10.4 (2.2)	

Results are reported as frequencies with percentages unless otherwise stated

IQR, interquartile range; SD, standard deviation

### Bivariate analysis of factors associated with the final place of care

Patients whose final place of care was home were significantly older, tended to be married or widowed, and most often had the availability of a caregiver in the form of paid help, compared to patients whose final place of care was the inpatient hospice or hospital (Table 2). Additionally, these patients, and their families, had most frequently indicated home as the preferred place of care and death. This group also had the highest proportion of patients with PPSv2 score < 40%, compared to patients whose final place of care was the inpatient hospice or hospital. Patients whose final place of care was home also had the lowest pain and depression scores compared to patients in the other two final places of care; however, they had the highest drowsiness scores (all *p* < 0.017).

**Table 2:** Significant factors associated with the final place of care in bivariate analysis

Variables	Final place of care			Adjusted P-value^***a***^
	Home (***N*** = 209)	Inpatient hospice (***N*** = 85)	Hospital (***N*** = 60)	
Age (years)				
Median (IQR)	79 (70–86)	71 (65–81)	71 (64–80)	< 0.0001^*b*^
Mean (SD)	77.7 (11.6)	71.6 (12.0)	72.3 (9.9)	< 0.0001^*c*^
Gender				
Male	96 (45.9)	46 (54.1)	39 (65.0)	0.252^*b*^
Female	113 (54.1)	39 (45.9)	21 (35.0)	0.014^*c*^
Marital status				
Single	8 (3.8)	18 (21.2)	6 (10.0)	
Married	100 (47.8)	38 (44.7)	40 (66.7)	
Divorced	7 (3.3)	9 (10.6)	2 (3.3)	<0.0001^*b*^
Widowed	94 (45.0)	20 (23.5)	12 (20.0)	0.003^*c*^
Housing type				
Rental	11 (5.3)	14 (16.5)	6 (10.0)	
Public	169 (80.9)	68 (80.0)	50 (83.3)	
Private condominium	15 (7.2)	2 (2.4)	2 (3.3)	0.002^*b*^
Private landed housing	14 (6.7)	1 (1.2)	2 (3.3)	0.300^*c*^
Government financial support level				
High	117 (56.0)	67 (78.8)	33 (55.0)	
Medium	20 (9.6)	7 (8.2)	9 (15.0)	
Low	23 (11.0)	5 (5.9)	7 (11.7)	0.001^*b*^
No support	49 (23.4)	6 (7.1)	11 (18.3)	0.605^*c*^
Cancer site				
Brain	6 (2.9)	1 (1.2)	2 (3.3)	
Head and neck	3 (1.4)	5 (5.9)	5 (8.3)	
Gastrointestinal system	54 (25.8)	27 (31.7)	14 (23.3)	
Hepatobiliary pancreatic system	36 (17.2)	15 (17.6)	14 (23.3)	
Breast	18 (8.6)	2 (2.4)	4 (6.7)	
Gynecological	3 (1.4)	0	0	
Genitourinary	12 (5.7)	5 (5.9)	3 (5.0)	
Hematological	10 (4.8)	2 (2.4)	1 (1.7)	
Prostate	5 (2.4)	2 (2.4)	3 (5.0)	
Skin	0	2 (2.4)	1 (1.7)	
Lung	50 (23.9)	17 (20.0)	8 (13.3)	
Multiple sites	4 (1.9)	4 (4.7)	2 (3.3)	
Unknown	8 (3.8)	1 (1.2)	2 (3.3)	0.024^*b*^
Others	0	2 (2.4)	1 (1.7)	0.001^*c*^
Cardiovascular disease	72 (34.4)	20 (23.5)	6 (10.0)	0.091^*b*^
				< 0.0001^*c*^
Dementia	35 (16.7)	5 (5.9)	1 (1.7)	0.023^*b*^
				0.005^*c*^
Main caregiver availability	207 (99.0)	61 (71.8)	53 (88.3)	< 0.0001^*b*^
Family	72 (34.8)	38 (62.3)	34 (64.2)	< 0.0001^*c*^
Friend	0	2 (3.3)	2 (3.8)	
Foreign domestic helper	129 (62.3)	21 (34.4)	15 (28.3)	< 0.0001^*b*^
Private/interim care nurse	6 (2.9)	0	2 (3.8)	< 0.0001^*c*^
Family caregiver age (years)				
Median (IQR)	54 (47–62)	61.5 (50.5–69)	51.5 (45–65)	0.007^*b*^
Mean (SD)	53.6 (12.8)	58.5 (13.7)	54.2 (14.6)	0.932^*c*^
Patient’s PPOC	138 (66.0)	70 (82.4)	41 (68.3)	
Home	135 (97.8)	31 (44.3)	21 (51.2)	
Inpatient hospice	1 (0.7)	38 (54.3)	3 (7.3)	
Hospital	1 (0.7)	0	16 (39.0)	
No preference	0	1 (1.4)	0	< 0.0001^*b*^
Any institution	1 (0.7)	0	1 (2.4)	< 0.0001^*c*^
Patient’s PPOD	136 (65.1)	70 (82.4)	35 (58.3)	
Home	130 (95.6)	33 (47.1)	25 (71.4)	
Inpatient hospice	1 (0.7)	34 (48.6)	5 (14.3)	
Hospital	1 (0.7)	0	2 (5.7)	
No preference	3 (2.2)	3 (4.3)	1 (2.9)	< 0.0001^*b*^
Any institution	1 (0.7)	0	2 (5.7)	< 0.0001^*c*^
Family’s PPOC	207 (99.0)	72 (84.7)	47 (78.3)	
Home	202 (97.6)	7 (9.7)	7 (14.9)	
Inpatient hospice	3 (1.4)	65 (90.3)	14 (29.8)	
Hospital	2 (1.0)	0	23 (48.9)	
No preference	0	0	1 (2.1)	< 0.0001^*b*^
Any institution	0	0	2 (4.3)	< 0.0001^*c*^
Family’s PPOD	206 (98.6)	72 (84.7)	42 (70.0)	
Home	196 (95.1)	8 (11.1)	16 (38.1)	
Inpatient hospice	4 (1.9)	63 (87.5)	14 (33.3)	
Hospital	2 (1.0)	0	8 (19.0)	
No preference	4 (1.9)	1 (1.4)	2 (4.8)	< 0.0001^*b*^
Any institution	0	0	2 (4.8)	< 0.0001^*c*^
Duration of enrollment in the home-based care service (days)				
Median (IQR)	59 (32–124)	92 (57–168)	63 (42.5–160)	< 0.0001^*b*^
Mean (SD)	93.7 (93.0)	130.1 (103.3)	107.6 (100.7)	0.080^*c*^
Number of emergency department visits				
Median (IQR)	0	0 (0–1)	0 (0–1)	< 0.0001^*b*^
Mean (SD)	0.3 (0.8)	0.7 (1.0)	0.7 (1.4)	0.054^*c*^
Number of hospital admissions				
Median (IQR)	0 (0–1)	1 (0–1)	0 (0–1)	< 0.0001^*b*^
Mean (SD)	0.4 (0.8)	0.9 (1.0)	0.8 (1.7)	0.196^*c*^
Average length of hospitalization (days)				
Median (IQR)	0 (0–4)	5 (0–11)	0 (0–5.5)	< 0.0001^*b*^
Mean (SD)	2.8 (6.2)	8.8 (11.0)	4.5 (9.4)	0.205^*c*^
PPSv2 2 weeks before death or admission to the final place of care				
10%	77 (36.8)	0	0	
20%	45 (21.5)	3 (3.5)	4 (6.7)	
30%	45 (21.5)	14 (16.5)	7 (11.7)	
40%	21 (10.0)	25 (29.4)	11 (18.3)	
50%	8 (3.8)	19 (22.4)	22 (36.7)	
60%	4 (1.9)	4 (4.7)	8 (13.3)	
70%	1 (0.5)	2 (2.4)	1 (1.7)	
80%	0	0	1(1.7)	<0.0001^*b*^
Missing	8 (3.8)	18 (21.2)	6 (10.0)	<0.0001^*c*^
ESASr 2 weeks before death or admission to the final place of care				
Pain				
Median (IQR)	0 (0–2)	0 (0–3)	2.5 (0–3)	< 0.0001^*b*^
Mean (SD)	0.9 (1.7)	2.2 (2.7)	2.1 (2.4)	<0.0001^*c*^
Nausea				
Median (IQR)	0	0	0	0.007^*b*^
Mean (SD)	0.2 (0.9)	0.6 (1.5)	0.6 (1.7)	0.047^*c*^
Depression				
Median (IQR)	0	0	0	< 0.0001^*b*^
Mean (SD)	0.2 (0.8)	1.1 (2.1)	0.9 (2.2)	0.001^*c*^
Drowsiness				
Median (IQR)	3 (0–8)	0 (0–5)	0 (0–3)	0.002^*b*^
Mean (SD)	4.1 (3.8)	2.5 (3.1)	2.0 (2.8)	< 0.0001^*c*^
Appetite				
Median (IQR)	5 (0–9)	5 (3–6.5)	3 (0–5)	0.608^*b*^
Mean (SD)	4.6 (3.9)	4.3 (3.1)	3.1 (2.8)	0.015^*c*^
Well-being				
Median (IQR)	0 (0–3)	3 (0–6)	3 (0–5)	0.001^*b*^
Mean (SD)	1.8 (2.5)	3.1 (2.9)	2.6 (2.7)	0.028^*c*^

Results are reported as frequencies with percentages unless otherwise stated

IQR, interquartile range; SD, standard deviation; PPOC, preferred place of care; PPOD, preferred place of death; PPSv2, Palliative performance scale v2 is a valid and reliable tool ranging from 0% (death) to 100% (normal function) for assessing the functional status of palliative care patients [36, 37]; ESASr, Edmonton symptom assessment scale is a psychometrically tested tool that uses a numeric rating scale ranging from 0 (no symptom) to 10 (worst) for measuring the symptom severity of nine symptoms (pain, fatigue, nausea, depression, anxiety, drowsiness, appetite, well-being and dyspnea). Additional symptoms can be recorded if present. Individual symptom scores are summed with a higher total score indicating worse symptom burden [38, 39]

^*a*^To control for type 1 error in multiple comparisons, Bonferroni adjustment was applied to the *p*-value. With three pairwise comparisons, the adjusted *p*-value for statistical significance was 0.017

^*b*^Adjusted p-value when patients whose final place of care was in the inpatient hospice were compared to patients whose final place of care was at home

^*c*^Adjusted p-value when patients whose final place of care was in the hospital were compared to patients whose final place of care was at home

Compared to patients in the home group, family caregivers of patients whose final place of care was the inpatient hospice were significantly older. There was also a higher proportion of patients in the inpatient hospice group who lived in rental housing, and received the highest level of government financial support. Patients in the inpatient hospice group had a significantly greater utilization of acute healthcare resources, and a significantly longer median enrollment duration in the home-based palliative care service, compared to patients in the home group. Nausea and well-being scores were significantly worse in the inpatient hospice group, compared to the home group (all *p* < 0.017).

There was a significantly higher proportion of males amongst patients whose final place of care was the hospital, compared to those who received care at home. However, there were fewer patients with lung cancer, cardiovascular disease, and dementia. Appetite problems were also less severe in the hospital group, compared to the home group (all *p* < 0.017) (Table 2).

### Multivariate logistic regression of factors associated with the final place of care

Marital status of patient, age of patient’s family caregiver, PPSv2 score, and patient’s place of death preference, predicted inpatient hospice as the final place of care in the binomial model (Additional file 1: Appendix Table A1). In the multinomial model, pain score was found to be an additional significant predictor of inpatient hospice as the final place of care. Patients were more likely to have received terminal care in the inpatient hospice if they were single or divorced (OR: 5.5; 95% CI: 1.1–27.8), compared to married or widowed patients; had older family caregivers (≥ 55 years) (OR: 3.1; 95% CI: 1.1–8.8), compared to those with younger family caregivers; had a PPSv2 score ≥ 40% (OR: 9.1; 95% CI: 3.3–24.8), compared to patients with lower functional status; had a pain score ≥ 2 (OR: 3.6; 95% CI: 1.3–9.8), compared to patients with lower pain scores; and, had expressed preference for a non-home place of death (OR: 23.8; 95% CI: 5.4–105.1), compared to patients with a home death preference (all *p* < 0.05) (Table 3).

**Table 3:** Multivariate analysis of factors associated with the final place of care using multinomial logistic regression

Variables	Inpatient hospice		Hospital	
	Adjusted odds ratio (95% CI)	P-value	Adjusted odds ratio (95% CI)	P-value
Gender				
Female	1.38 (0.52–3.63)		1	
Male	1	0.518	3.16 (1.01–9.90)	0.048
Marital status				
Married/widowed	1		1	
Single/divorced	5.52 (1.10–27.78)	0.038	5.00 (0.92–27.03)	0.063
Family caregiver age				
< 55 years	1		1.30 (0.43–3.91)	
≥55 years	3.05 (1.06–8.78)	0.038	1	0.638
PPSv2				
< 40%	1		1	
≥40%	9.10 (3.34–24.82)	<0.0001	8.64 (2.87–26.00)	<0.0001
Pain				
< 2	1		1	
≥2	3.61 (1.33–9.79)	0.012	3.45 (1.16–10.27)	0.026
Patient’s PPOD				
Home	1		1	
Non-home	23.76 (5.37–105.08)	< 0.0001	9.77 (2.07–46.25)	0.004

Reference category: home

CI, confidence interval; PPSv2, Palliative performance scale v2 is a valid and reliable tool ranging from 0% (death) to 100% (normal function) for assessing the functional status of palliative care patients [36, 37]; PPOD, Preferred place of death

Gender, PPSv2 score, pain score, and patient’s place of death preference, predicted hospital as the final place of care in both the binomial (Additional file 1: Appendix Table A2) and multinomial models. Patients were more likely to have received terminal care in the hospital if they were a male (OR: 3.2; 95% CI: 1.0–9.9), compared to being a female; had PPSv2 score ≥ 40% (OR: 8.6; 95% CI: 2.9–26.0), compared to patients with lower functional status; had a pain score ≥ 2 (OR: 3.5; 95% CI: 1.2–10.3), compared to those with lower pain scores; and, had expressed preference for a non-home place of death (OR: 9.8; 95% CI: 2.1–46.3), compared to those who preferred home death (all *p* < 0.05) (Table 3).

### Goal-concordance and congruence

Despite substantial agreement between patients’ preferences for places of care and death (90.4%, kappa = 0.75), goal-concordance with their preferred place of care was moderate (76.7%, kappa = 0.54), while goal-concordance with their preferred place of death was fair (72.6%, kappa = 0.39). In contrast, goal-concordance with families’ preferences for places of care and death were substantial (89.9%; kappa = 0.79 and 86.3%; kappa = 0.67, respectively). Congruencies between patients’ and their families’ preferences were moderate (81.4%; kappa = 0.59 and 80.4%; kappa = 0.55 for place of care and death preferences, respectively).

### Post-bereavement measures

Families of patients whose final place of care was the inpatient hospice required professional bereavement support more frequently (30.6%) than those of patients whose final place of care was the hospital (26.7%) or home (21.5%) (*p* < 0.0001). However, there was no difference in the level of death acceptance by families across the three care settings (*p* = 0.869).

## Discussion

This study sought to examine factors associated with the final place of care of patients with advanced cancer receiving integrated, home-based palliative care. Single or divorced patients, and those with older family caregivers were more likely to receive terminal care in the inpatient hospice, while male patients were more likely to do so in the hospital. Higher functional status, greater pain severity, and patient preference for non-home death, were associated with both the inpatient hospice and hospital as the final places of care. Goal-concordance with patients’ preferences was fair to moderate, while patients’ and their families’ wishes were moderately congruent. However, unlike other studies, socioeconomic status [10, 42] was not found to be an independent predictor of the final place of care in this study. This could be due to the substantial subsidies provided by the Singapore healthcare financing system for home-based palliative care, inpatient acute care, and inpatient hospice services. Coupled with the national healthcare insurance and savings schemes [43], the out-of-pocket payments required among the different settings may be fairly comparable. Acute healthcare utilization [13, 32, 44] was also not found to be an independent predictor of the final place of care, possibly due to the home-based team’s regular contact with, and prompt response to, patients’ needs throughout their illness trajectories [26].

In the Asian context, children and spouses are bound by cultural values and societal norms to care for their immediate relatives during periods of illness [13]. Thus, marital status was a predictor of inpatient hospice as the final place of care, as single or divorced patients lack the social support that is traditionally accorded through marriage [8]. While other studies have found living arrangement to be predictive of the place of death [8, 11, 13, 24], it was not observed in this study. Both living arrangement and marital status are surrogate measures of social support, and the latter could be a more direct indicator than the former. Additionally, many families depend on paid help to provide direct hands-on care [13]. Hence, differences in living arrangements may not be that instrumental in influencing the final place of care in a small and well-connected country like Singapore, compared to other countries.

Family caregivers play a vital role in the care of patients with advanced cancer in the home setting. They supplement the professional care provided by the home-based team, and informal care provided by paid help [45]. Long-term care of functionally dependent patients with cancer can be particularly demanding, both physically and psychologically, especially on older caregivers [22]. Over time, their ability to provide care at home safely may be compromised, which then necessitates inpatient hospice admission of their loved ones for terminal care [45]. This may explain the effect that family caregiver’s age has on predicting the final place of care. In our study, male patients were more likely to be hospitalized for terminal care. This is consistent with the findings of other similar studies [13, 44], and may possibly be due to their higher likelihood of receiving chemotherapy near the end-of-life [46].

The final place of care of patients with a higher functional status were more likely to be in the inpatient hospice or hospital, compared to bed-bound and fully assisted patients, consistent with the findings of systematic reviews [8, 11]. Patients who have better functionality (in terms of ambulation and activity levels) and alertness may strive to maintain their status through hospital interventions [4], while patients with PPSv2 scores between 40 and 60%, and who are not as drowsy, may require a level of supervision and physical assistance beyond caregivers’ coping capacity, thereby predisposing them to inpatient hospice admission for terminal care. Qualitative analysis of the reasons for admission to the inpatient hospice and hospital may provide more insights into this finding. Given the better functional status of such patients, the incorporation of hospice daycare into the integrated model to supplement care for patients who prefer to remain at home may be considered.

Pain severity also predicted inpatient hospice and hospital as the final places of care. Complex pain, and its associated psychological implications, may be stressful for caregivers to manage on their own at home, which can affect care quality [11, 14, 16–19]. To ensure comfort at the end-of-life, patients and their families may opt for an institutional setting where professional care and interventions are readily available [45].

Patient’s preference for non-home death was the strongest predictor of inpatient hospice and hospital as the final places of care which is consistent with findings from other studies [8, 10, 11, 24, 44]. The home-based team initiated care planning discussions with patients and their families at an early stage, and revised them as and when circumstances changed during the course of the patient’s illness. These discussions were documented in the electronic medical records which allowed all healthcare professionals involved in their care to remain apprised of their preferences. Hence, they were able to work collaboratively to support and meet patients’ and their families’ preferences as much as possible [26]. The utility of such care planning discussions is reflected in the moderate congruence between patients’ and their families’ preferences, as well as the substantial level of goal-concordance with families’ preferences. However, the sub-optimal level of goal-concordance with patients’ preferences found in this study has implications for policy and practice.

### Implications of findings

Although close to 60% of our patients died at home which was above the national average of 28% in Singapore [44], the sub-optimal level of concordance with patients’ preferences observed revealed gaps within the integrated model that requires addressing at the national, organizational, and individual levels [25]. More comprehensive social networks in the community could be established to support single or divorced patients who wish to remain at home at the end-of-life [44, 47]. The influence of family-related factors on a patient’s final place of care, and the disparity in goal-concordance with patients’ compared to their families’ preferences, highlighted the instrumental role that families play in the end-of-life care of patients with advanced cancer. Hence, more practical and emotional support could be provided to better equip families for the care and death of patients who prefer to be at home [4, 13, 21, 29]. Such support would serve to mitigate potential psychosocial sequelae. Patients whose final places of care were in institutions reported lower mood and well-being, and a higher proportion of their families required professional bereavement support. In addition, home was not the preferred place of care and death for a substantial minority (~ 20%) of patients. Given the vital role that the integrated model plays in catering to the diverse preferences and needs of patients and their families, additional funding and capacity could be provided to further enhance the model [24, 26].

Although pain was not the most severe symptom, a score of 2 was sufficient to predict institutionalization. In order to keep patients who prefer to remain at home comfortable during their end-of-life, analgesia could be pre-emptively prescribed to optimize pain control, after due deliberation has been given regarding the propensity for misuse [48]. Caregivers should also be adequately trained on its complex administration. Additionally, being cognizant of the predictors of the final place of care allows care planning discussions to be personalized, thereby improving communication and care satisfaction when preferences are met [11, 32, 49]. Apart from allowing realistic goals to be set, transitions between care settings can also be arranged in a timely manner. This prevents traumatic changes at the end-of-life, and minimizes the risk of complicated grief [4, 50]. The high rates of care planning discussions with patients (~ 70%) and their families (~ 90%) observed in this study suggest that these important conversations were taking place in practice, but efforts should continue to initiate them early [3, 4]. However, when faced with non-modifiable factors, the wishes of functionally better male patients for hospital interventions should be respected.

### Strengths and limitations

This is one of the few studies examining factors associated with the final place of care, among patients with advanced cancer who were receiving home-based palliative care within an integrated model. As care planning is dynamic, the last discussion prior to death was obtained to capture the final wishes of patients. Additionally, information relating to symptoms and functional status 2 weeks prior to death, or admission to the final place of care, were collected to better reflect patients’ conditions at the end-of-life, overcoming the limitations of previous studies [10, 24].

However, this study had several limitations. Causal links could not be established from associations identified, and coding independent variables as binary indicators could have affected the performance of the multivariate model. Although intensity of home-based palliative care service was associated with the place of death in some studies [8, 13, 24], it was not investigated in this study. Additionally, not all patients had discussed or expressed their care preferences which would result in selection bias. Hence, the goal-concordance and congruence outcomes should be interpreted cautiously. Due to the retrospective study design, some inferences made about patients’ and their families’ preferences could not be confirmed. However, attempts were made to overcome this by reviewing the documentation of all healthcare professionals involved in the patients’ care for corroboration. Even though the findings may have limited generalizability to societies with differing cultural norms, values and healthcare systems, it may potentially still be applicable to similar patient populations and care settings. Due to different care needs and disease trajectories [51], the findings in this study involving patients with advanced cancer cannot be extrapolated to patients with non-cancer diseases. Future studies involving patients with non-cancer diseases may be required for providing insights into the elements needed to meet the needs and preferences of specific patient populations for the final place of care.

## Conclusions

Higher functional status, greater pain intensity, and preference for non-home death, predicted institutionalization as the final place of care. Additionally, single or divorced patients with older family caregivers were more likely to receive terminal care in the inpatient hospice, while males were more likely to be hospitalized. Despite being part of an integrated care model, concordance with patients’ preferences was sub-optimal. Policy makers could consider establishing more comprehensive community networks for patients with poor social support, while additional resources may be allocated to support families caring for patients who wish to remain at home. Optimizing pain management, and more personalized care planning discussions, are also recommended in practice to improve the likelihood of meeting patients’ preferences for the final place of care. Future research should similarly examine factors associated with the final place of care in patients with advanced non-cancer conditions.

## Supplementary Information

**Additional file 1.** Appendix Tables A1 and A2.

## Data Availability

The datasets used and analyzed during this study are available from the corresponding author on reasonable request.

## Abbreviations

PPSv2: Palliative Performance Scale v2
ESASr: Edmonton Symptom Assessment System
