# Supplementary material for: Predictors of the final place of care of patients with advanced cancer receiving integrated home-based palliative care: a retrospective cohort study
Source: BMC Palliat Care. 2021 Oct 18;20:164. doi: 10.1186/s12904-021-00865-5 (PMC8522009; doi:10.1186/s12904-021-00865-5)
Supplement: Supplementary file 1 — Additional file 1. Appendix Tables A1 and A2. [file 12904_2021_865_MOESM1_ESM.docx]

| **Table A1** Multivariate analysis of factors associated with inpatient hospice as the final place of care compared to home using binomial logistic regression | | |
| --- | --- | --- |
| **Variables** | **Adjusted odds ratio**  **(95% CI)** | **P-value** |
| Age |  | 0.868 |
| <75 years | 1 |  |
| ≥75 years | 1.12 (0.31-4.08) |  |
| Marital status |  | 0.018 |
| Married/widowed | 1 |  |
| Single/divorced | 15.20 (1.59-145.08) |  |
| Government financial support level |  | 0.080 |
| Other levels | 1 |  |
| Highest level | 3.74 (0.85-16.39) |  |
| Family caregiver age |  | 0.011 |
| <55 years | 1 |  |
| ≥55 years | 8.00 (1.61-40.00) |  |
| Main caregiver |  | 0.841 |
| Family/friend | 1 |  |
| Paid help*^a^* | 1.17 (0.25-5.38) |  |
| Duration of enrollment in homecare |  | 0.219 |
| <60 days | 1 |  |
| ≥60 days | 2.49 (0.58-10.75) |  |
| Average length of hospitalization |  | 0.365 |
| <3 days | 1 |  |
| ≥3 days | 1.87 (0.48-7.25) |  |
| PPSv2 |  | 0.001 |
| <40% | 1 |  |
| ≥40% | 10.10 (2.51-40.00) |  |
| Pain |  | 0.053 |
| <2 | 1 |  |
| ≥2 | 3.39 (0.98-11.76) |  |
| Drowsiness |  | 0.487 |
| <3 | 1 |  |
| ≥3 | 1.56 (0.44-5.49) |  |
| Well-being |  | 0.174 |
| <3 | 1 |  |
| ≥3 | 2.33 (0.69-7.87) |  |
| Patient’s PPOD |  | 0.001 |
| Home | 1 |  |
| Non-home | 34.50 (4.10-333.30) |  |
| CI, confidence interval; PPSv2, Palliative performance scale v2 is a valid and reliable tool ranging from 0% (death) to 100% (normal function) for assessing the functional status of palliative care patients;^(36, 37)^ PPOD, Preferred place of death.  *^a^*Paid help includes foreign domestic helper engaged primarily to care for patient and private or interim care nurse. | | |

| **Table A2** Multivariate analysis of factors associated with hospital as the final place of care compared to home using binomial logistic regression | | |
| --- | --- | --- |
| **Variables** | **Adjusted odds ratio**  **(95% CI)** | **P-value** |
| Age |  | 0.991 |
| <75 years | 1 |  |
| ≥75 years | 1.01 (0.33-3.05) |  |
| Gender |  | 0.024 |
| Female | 1 |  |
| Male | 3.71 (1.18-11.61) |  |
| Marital status |  | 0.678 |
| Married/widowed | 1 |  |
| Single/divorced | 1.49 (0.23-9.94) |  |
| Main caregiver |  | 0.405 |
| Family/friend | 1.61 (0.53-4.90) |  |
| Paid help*^a^* | 1 |  |
| Cancer site |  | 0.609 |
| Gastrointestinal, hepatobiliary pancreatic, genitourinary system, prostate, lung | 1 |  |
| Others | 1.38 (0.40-4.74) |  |
| PPSv2 |  | 0.002 |
| <40% | 1 |  |
| ≥40% | 5.99 (1.95-18.52) |  |
| Pain |  | 0.041 |
| <2 | 1 |  |
| ≥2 | 2.97 (1.05-8.40) |  |
| Drowsiness |  | 0.458 |
| <3 | 1 |  |
| ≥3 | 1.57 (0.48-5.21) |  |
| Appetite |  | 0.538 |
| <4 | 1 |  |
| ≥4 | 0.71 (0.24-2.10) |  |
| Patient’s PPOD |  | 0.010 |
| Home | 1 |  |
| Non-home | 7.19 (1.60-32.26) |  |
| CI, confidence interval; PPSv2, Palliative performance scale v2 is a valid and reliable tool ranging from 0% (death) to 100% (normal function) for assessing the functional status of palliative care patients;^(36, 37)^ PPOD, Preferred place of death.  *^a^*Paid help includes foreign domestic helper engaged primarily to care for patient and private or interim care nurse. | | |
